# Antibacterial and Antiviral Properties of Chenopodin-Derived Synthetic Peptides

**DOI:** 10.3390/antibiotics13010078

**Published:** 2024-01-14

**Authors:** Marcia L. Feijoo-Coronel, Bruno Mendes, David Ramírez, Carlos Peña-Varas, Nina Q. E. de los Monteros-Silva, Carolina Proaño-Bolaños, Leonardo Camilo de Oliveira, Diego Fernandes Lívio, José Antônio da Silva, José Maurício S. F. da Silva, Marília Gabriella A. G. Pereira, Marina Q. R. B. Rodrigues, Mauro M. Teixeira, Paulo Afonso Granjeiro, Ketan Patel, Sakthivel Vaiyapuri, José R. Almeida

**Affiliations:** 1 Biomolecules Discovery Group, Universidad Regional Amazónica Ikiam, Km 7 Via Muyuna, Tena 150101, Ecuador; 2 Departamento de Farmacología, Facultad de Ciencias Biológicas, Universidad de Concepción, Concepción 4030000, Chile; 3 Centro de Pesquisa e Desenvolvimento de Fármacos, Departamento de Bioquímica e Imunologia, Instituto de Ciências Biológicas, Federal University of Minas Gerais, Belo Horizonte 31270-901, Brazil; 4 Campus Centro Oeste, Federal University of São João Del-Rei, Rua Sebastião Gonçalves Filho, n 400, Chanadour, Divinópolis 35501-296, Brazil; 5 Departamento de Bioquímica, Centro de Ciências Biomédicas, Federal University of Alfenas, Rua Gabriel Monteiro da Silva, 700, Sala E209, Alfenas 37130-001, Brazil; 6 Departamento de Engenharia de Biossistemas, Campus Dom Bosco, Federal University of São João Del-Rei, Praça Dom Helvécio, 74, Fábricas, São João del-Rei 36301-160, Brazil; 7 School of Biological Sciences, University of Reading, Reading RG6 6UB, UK; 8 School of Pharmacy, University of Reading, Reading RG6 6UB, UK

**Keywords:** antimicrobial, antiviral, Chenopodin, membranolytic, quinoa, synthetic peptides

## Abstract

Antimicrobial peptides have been developed based on plant-derived molecular scaffolds for the treatment of infectious diseases. Chenopodin is an abundant seed storage protein in quinoa, an Andean plant with high nutritional and therapeutic properties. Here, we used computer- and physicochemical-based strategies and designed four peptides derived from the primary structure of Chenopodin. Two peptides reproduce natural fragments of 14 amino acids from Chenopodin, named Chen1 and Chen2, and two engineered peptides of the same length were designed based on the Chen1 sequence. The two amino acids of Chen1 containing amide side chains were replaced by arginine (ChenR) or tryptophan (ChenW) to generate engineered cationic and hydrophobic peptides. The evaluation of these 14-mer peptides on *Staphylococcus aureus* and *Escherichia coli* showed that Chen1 does not have antibacterial activity up to 512 µM against these strains, while other peptides exhibited antibacterial effects at lower concentrations. The chemical substitutions of glutamine and asparagine by amino acids with cationic or aromatic side chains significantly favoured their antibacterial effects. These peptides did not show significant hemolytic activity. The fluorescence microscopy analysis highlighted the membranolytic nature of Chenopodin-derived peptides. Using molecular dynamic simulations, we found that a pore is formed when multiple peptides are assembled in the membrane. Whereas, some of them form secondary structures when interacting with the membrane, allowing water translocations during the simulations. Finally, Chen2 and ChenR significantly reduced SARS-CoV-2 infection. These findings demonstrate that Chenopodin is a highly useful template for the design, engineering, and manufacturing of non-toxic, antibacterial, and antiviral peptides.

## 1. Introduction

Infectious diseases represent a serious and challenging problem in modern medicine, with significant economic and health impacts globally [1,2]. Until 2023, the SARS-CoV-2 virus affected around 773 million people and caused approximately 6.9 million deaths [3]. Notably, 4 to 15% of hospitalised patients acquired bacterial infections that increase the mortality rate [4,5,6]. In an era of constant global change, the emergence of new diseases, such as COVID-19, large-scale outbreaks and multidrug-resistant bacteria constitute a significant medical issue that requires innovative solutions [7,8]. Several naturally occurring compounds, particularly from plant-based proteins, and peptides have successfully assisted in tackling infectious diseases [9,10]. Therefore, a bio-prospecting approach is likely to result in the identification of additional compounds to treat infectious and other human diseases, such as hypertension, diabetes, cancer, atherosclerosis, and Parkinson’s disease.

According to the Food and Agriculture Organization of the United Nations (FAO), total proteins in quinoa provide more than 180% of the required levels of nine essential amino acids, which cannot be synthesised in human cells and play an important role in maintaining good health [11]. Some proteins and peptides present in quinoa have been recognised for their antioxidant, antidiabetic, antihypertensive, and anti-bacterial properties [12,13]. Lower molecular weight peptides generated during alcalase- and trypsin-mediated hydrolysis scavenge free radicals and increase antioxidant actions. On the other hand, some of these biomolecules act as natural inhibitors of α-glucosidase, which is responsible for carbohydrate absorption, and controlling metabolic conditions, such as postprandial hyperglycemia and type 2 diabetes mellitus [14,15].

The most abundant protein in quinoa seeds is Chenopodin, which is an oligomeric 11S globulin with lectin-like properties, a relative molecular mass of 320 kDa and an isoelectric point (pI) of 6.58. This protein represents 37% of the total protein contents in quinoa [16,17,18]. A previous study has demonstrated the antimicrobial properties of this protein against *Escherichia coli*, *Salmonella enterica* and *Pseudomonas aeruginosa* [19]. Other investigations have shown its anti-inflammatory, immunomodulatory and hemagglutination properties [16,20,21]. This multifunctional protein has a hexameric quaternary structure composed of subunits formed by α- and β-chains connected by a disulfide bridge [18,22]. These subunits differ in composition, length, size, and charge. The β-chain exhibits a low molecular weight and is rich in positively charged amino acids (so, it is also known as the basic subunit) [18].

Cationic lytic peptides inspired by molecular scaffolds from natural sources have been proposed as a basis for the development of next-generation antibiotics and antiviral agents [23,24]. Positively charged and hydrophobic amino acids, such as arginine, lysine, leucine, and tryptophan are key elements of membranolytic peptide-based drugs available in the market [25,26]. Historically, quinoa protein is identified as a promising precursor of several therapeutically valuable short peptides with health benefits [27]. These functional and biochemical characteristics turn quinoa proteins into potential candidates for the design of nutraceutical products and functional foods [28]. The discovery of therapeutic entities is a slow, long, and costly process originally based on wet-laboratory approaches [26]. However, this field has developed more rapidly due to the valuable assistance of computational/bioinformatics tools and the progress of artificial intelligence applications [29,30]. Therefore, in this study, four 14-mer peptide sequences were generated using computational design, based on the basic subunit of Chenopodin protein as a structural template, and their antibacterial and antiviral properties were evaluated.

## 2. Results

### 2.1. In Silico Design and Characterisation of Chenopodin-Derived Peptides

**Figure 1:**
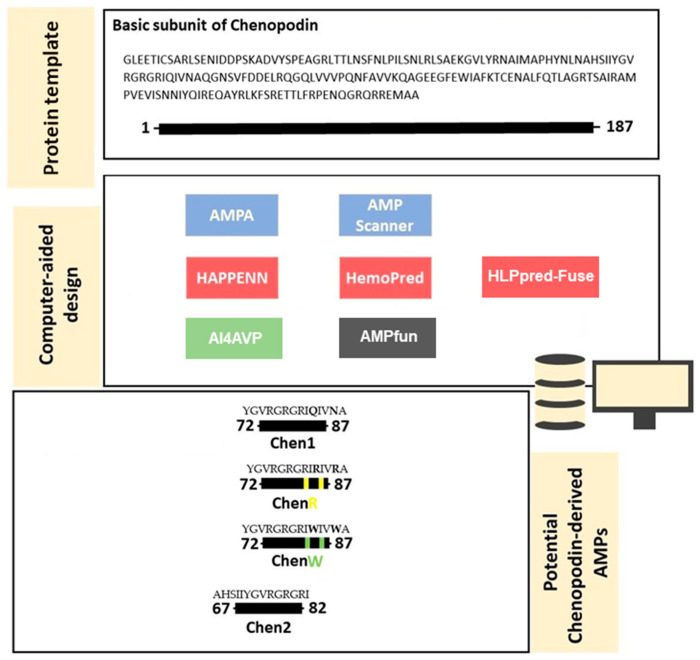
Antimicrobial and hemolytic screening of the primary structure of the basic subunit of Chenopodin using bioinformatic tools. In silico approaches employed to predict antibacterial, hemolytic, and antiviral activities are highlighted in blue, red, and green, respectively. AMPfun is coloured in grey because it was used to predict both antibacterial and antiviral properties. Most of these sequence-based predictors classify the peptide in antimicrobial/non-antimicrobial, hemolytic/non-hemolytic and antiviral/non-antiviral or show a column “score”, which indicates how much probability a peptide candidate displays a certain effect. According to AMPA software, this protein subunit has a potential antimicrobial region (72–87 segment—Chen1). We also selected another region (67–82 segment—Chen2) with potential antibacterial and antiviral actions and low haemolytic tendency. Two new peptide analogues (ChenR and ChenW) were designed by replacing two amino acids in Chen 1. Replacement sites in the sequence are highlighted in green and yellow. Yellow dots represent substitutions with arginine, while green symbolises that amino acids have been replaced by tryptophan. Overall, cationicity-enhanced and hydrophobicity-optimised analogues showed a higher probability of exerting antibacterial effects than Chen-1. The differences between the sequences of ChenR and ChenW peptides compared to Chen1 are highlighted in bold.

The bioinformatic analysis of the basic subunit of Chenopodin using AMPA software revealed a short region composed of 14 amino acid residues (72–87 segment), which can play a role as a molecular determinant for the antibacterial potential of the vegetal protein (Chenopodin). We named this sequence Chen1. Additionally, we selected the region 67–82 from Chenopodin and named it Chen2. The molecular region of Chen2 contains a partial sequence of Chen1; this has been identified as a potential antibacterial peptide by AMPfun and AMP Scanner. Cationic and hydrophobic optimisations are useful routes to fine-tuning the biological activity of peptide candidates. Therefore, two other peptides were constructed based on the primary structure of Chen1, in which asparagine at position 10 and glutamine at position 13 were replaced by either tryptophan (ChenW) or arginine (ChenR) (Figure 1). The computational screening predicted that these enhanced cationic and hydrophobic-optimised peptides are likely to be effective AMPs, with a high ability to affect Gram-positive and Gram-negative bacteria and viruses, and a low probability of inducing hemolytic activity (Supplementary Material Tables S1–S6).

Using in silico approaches, we estimated physicochemical properties of the computer-designed peptides evaluated in this study. These peptides have a positive charge, basic pI, and a high hydrophobicity score (Table 1). Peptides derived from Chenopodin (Chen1 and Chen2) possess lower molecular weights than engineered peptides. ChenR shows the highest charge (+6) and pI (13.09). These four peptides were synthesised, purified, characterised, and tested under in vitro experimental settings.

**Figure 2:**
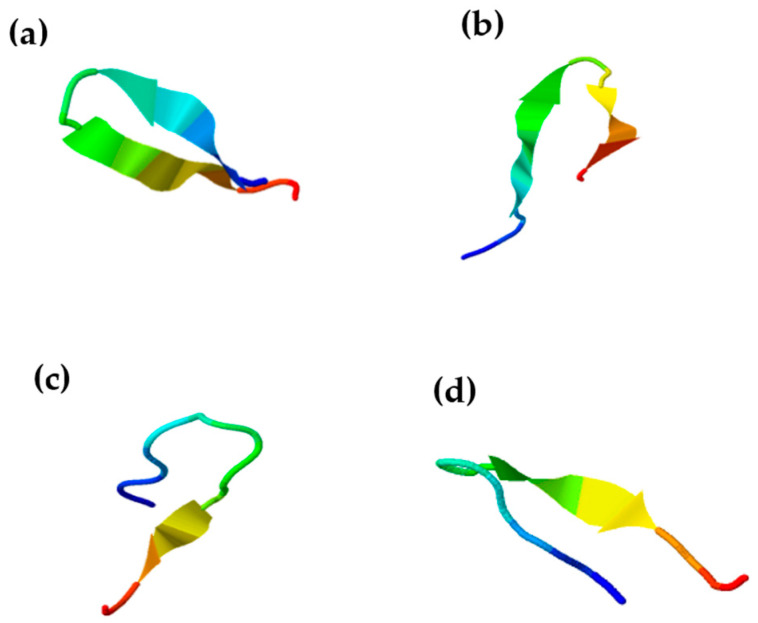
Predicted secondary structures of Chenopodin-derived peptides, (**a**) Chen 1, (**b**) Chen2, (**c**) ChenR, and (**d**) ChenW. The structures were predicted using the I-TASSER platform. PED2D predictor was also employed to confirm the suggested secondary structures. The results are shown in Supplementary Material Table S7.

Chenopodin-derived peptides did not show the classic alpha-helix structure exhibited by most AMPs. These peptides have a probability from 64.22% to 85% for acquiring a random coil secondary structure according to the PED2D predictor (Supplementary Material Table S7). In addition, the β-sheet structure is manifested in a lower percentage, which can be observed in the simulation shown by I-TASSER (Figure 2).

### 2.2. Synthesis and Characterisation of Chenopodin-Derived Peptides

**Figure 3:**
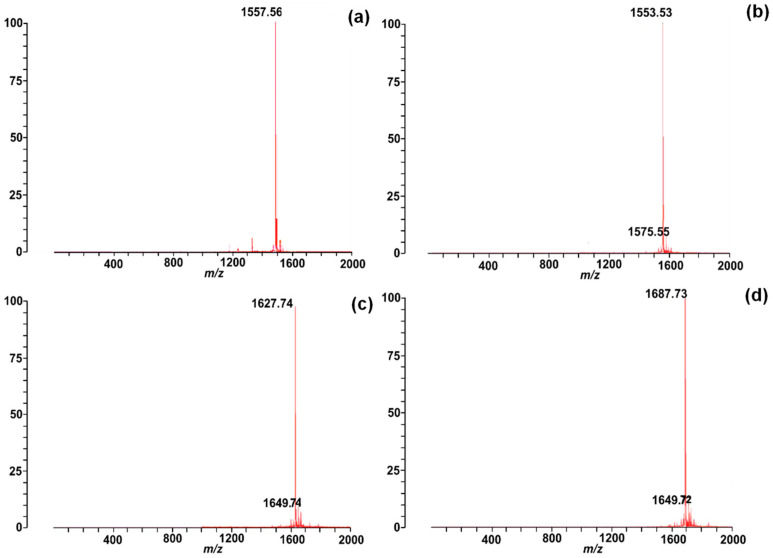
Mass spectra of Chenopodin-derived peptides: (**a**) Chen1, *m*/*z* = 1557.56 Da; (**b**) Chen2, *m*/*z* = 1553.53 Da; (**c**) ChenR, *m*/*z* = 1627.74 Da; (**d**) ChenW, *m*/*z* = 1687.73 Da, as identified through MALDI-ToF mass spectrometry.

The Chenopodin-derived peptides were chemically obtained using a standard Fmoc strategy. The peptides showed a high purity level (>90%) when assessed by the reversed-phase high-performance liquid chromatography (RP-HPLC). The analytical C18 RP-HPLC profiles of pure Chenopodin-derived peptides are shown in the Supplementary Material Figure S1**.** Synthetic peptides elute between 26 and 29 min. Their purity is also reflected in the mass spectrometry analysis of the peptides (Figure 3). The experimental masses of the peptides agree with the predicted values from the in silico analysis, which indicates the correct synthesis of peptides.

### 2.3. Chenopodin-Derived Peptides Exhibit Antibacterial Activities

The antibacterial properties of synthetic peptides were assessed by a broth dilution method and compared to a penicillin-type antibiotic that is routinely employed to manage a wide range of bacterial infections. At concentrations of ≤512 µM, Chen1 had no effects against *S. aureus* and *E. coli*. The remaining peptides (Chen2, ChenR, and ChenW) showed a range of minimum inhibitory concentrations (MIC) against Gram-positive (MIC: 8–128 µM) and Gram-negative (MIC: 8–64 µM) bacteria. Therefore, the antibacterial activity of the Chenopodin-derived peptides predicted by the in silico tools was confirmed, except for Chen1. Overall, ChenW showed a higher inhibition on bacteria growth, with MIC and MBC (minimum bactericidal concentration) values of 8 µM (Table 2).

### 2.4. Chenopodin-Derived Peptides Damage the Bacterial Cell Membrane

**Figure 4:**
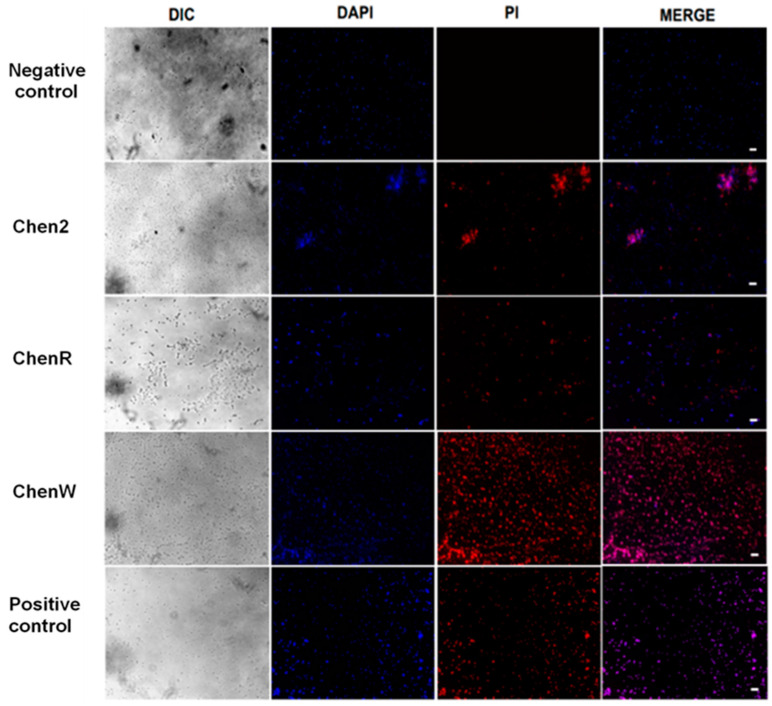
Chenopodin-derived peptides affect the bacterial membrane integrity. The bacterial membrane integrity was analysed using dual staining (DAPI/PI) by fluorescence micrographs on *S. aureus* incubated with Chen2, ChenR, and ChenW at 1× MIC. Untreated cells served as the negative control, and 10% (*v*/*v*) SDS was used as the positive control. The scale bars are 10 μm.

The assessment of cell membrane integrity was performed using dual-staining to better understand the impact of Chenopodin-derived peptides on bacteria. The results demonstrate that these synthetic peptides showed a membranolytic activity when analysed by fluorescence microscopy. *S. aureus* incubated with Chen2, ChenR, and ChenW showed an extensive uptake of propidium iodide (PI), as demonstrated through a high level of red fluorescence (Figure 4). The uptake of this red-fluorescent dye suggests the changes in cell membrane permeability caused by the peptides, similar to the membrane-disrupting action of detergents. ChenW induced the highest red fluorescence, like the effects observed in the positive control [10% (*v*/*v*) sodium dodecyl sulphate (SDS)]. Untreated bacterial cells did not show any membrane damage and retained the blue colour corresponding to 4′,6-diamidino-2-fenilindol (DAPI).

### 2.5. Interactions between ChenW and Bacterial Membranes

**Figure 5:**
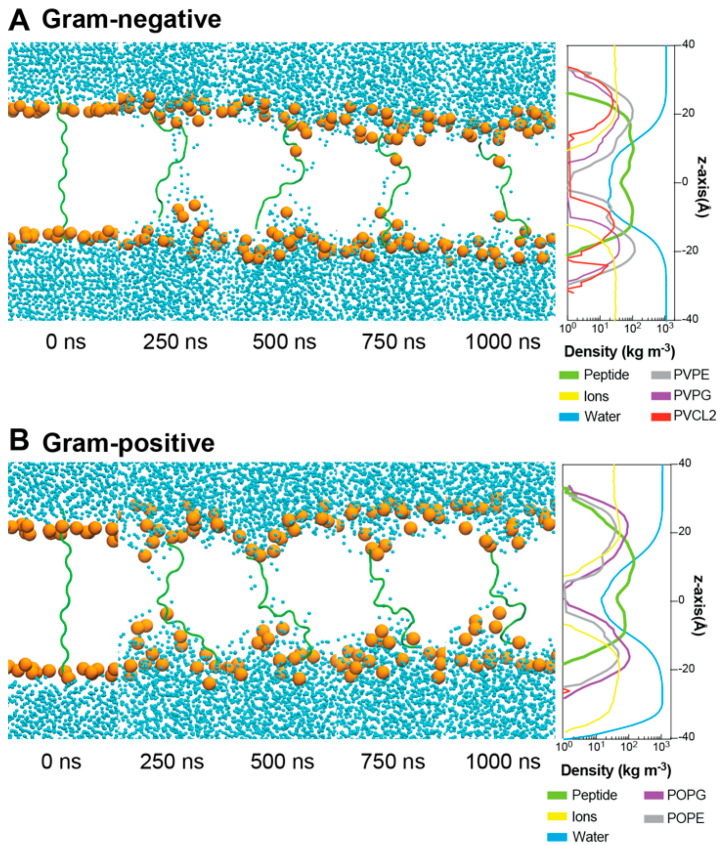
Unbiased molecular dynamic simulations of ChenW peptide embedded in Gram-negative (**A**) and Gram-positive (**B**) membranes reveal spontaneous water translocation. For better representation, only phosphate groups (orange), water (blue), and peptides (green) are displayed through the 1000 ns-MDs.

To determine how ChenW peptide (which showed a higher antibacterial activity) interacts with Gram-positive and Gram-negative membranes, molecular dynamic simulations (MDs) were performed. The peptide was modelled without an associated secondary structure and studied while a secondary structure was formed upon interaction with the membranes. After more than 1 µs-MDs, no conformational change in the secondary structure was evidenced, which is typical of some peptides with similar properties [31,32,33,34]. Then, MD simulations of the ChenW peptide were performed directly in Gram-negative (PVPE:PVPG:PVCL2—ratio 75:20:5) and Gram-positive (POPE:POPG—ratio 75:25) membranes. For this purpose, systems were constructed with the peptide inserted vertically into the lipid membrane (Figure 5). After minimisation and equilibration, 1 µs-MDs were performed for ChenW in each type of membrane. For both systems, it is observed that no secondary structure is formed during the simulation and that the peptide remains in a random-coil conformation. Furthermore, it is possible to observe that after 250 ns (and during the whole simulation) there is a translocation of water molecules and deformation of the membrane in the vicinity of the peptide, a phenomenon that has been evidenced in other peptides when simulating with different biological membranes [33]. Both density profiles allow us to quantify water translocation as well as peptide stability between both membrane layers.

**Figure 6:**
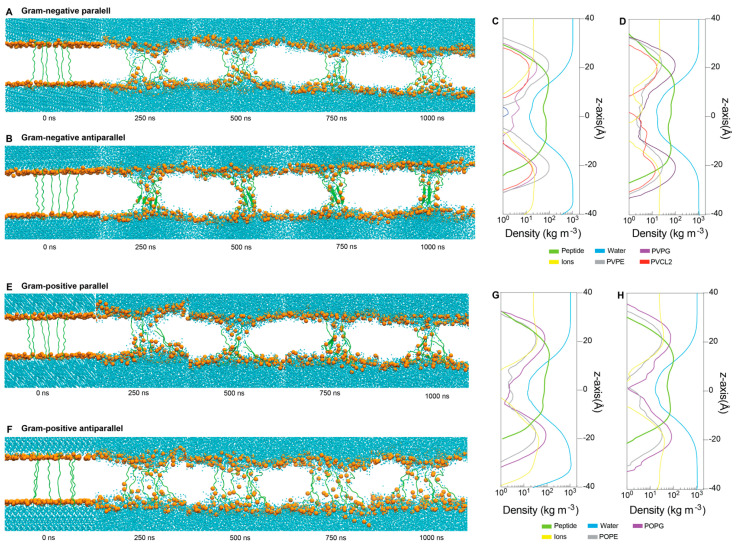
Unbiased molecular dynamics simulations of multiple (six) ChenW peptides embedded in Gram-negative (**A**,**B**) and Gram-positive (**E**,**F**) membranes reveal spontaneous water translocation. Density profiles for Gram-negative membranes and ChenW peptides in parallel (**C**) and antiparallel (**D**) conformations, as well as Gram-positive membranes and peptides in parallel (**G**) and antiparallel (**H**) conformations. For better representation, only phosphate groups (orange), waters(blue), and the peptides (green) are displayed through the 1000 ns-MDs.

To explore how multiple ChenW peptides interact with both Gram-negative and Gram-positive membranes, we constructed two different peptide set-ups; the first with six ChenW peptides located vertically in the same direction, meaning with the N-terminal facing towards the inner layer of the membrane (parallel), and the second with six ChenW peptides also located vertically but this time in alternate directions, meaning with the N-terminal domain of three peptides facing towards the inner layer of the membrane, and the other three peptides N-terminal domain facing towards the outer layer of the membrane (Figure 6A). It was possible to identify all systems after 250 ns water translocation, membrane deformations, and thus pore formation. The ChenW antiparallel peptides interacted with the Gram-negative membrane from the β-sheet secondary structure during the simulations, after 250 ns and during the entire trajectory (Figure 6B). A lower degree of β-sheet formation was also displayed in the ChenW parallel peptides interacting with the Gram-positive membrane (Figure 6E). The other two systems did not show a secondary structure formation. These results suggest that when the ChenW peptides assemble on the membrane (interacting with other ChenW peptides) they can stably structure themselves forming β-sheets and thus the stable pore, as has been observed with other pore-forming peptides by molecular dynamics studies [35]. We suggest that the same pattern was not observed in all simulations due to the limited conformational sampling that can be performed in 1 µs-MD simulation. Hence, we plan to achieve longer MDs and increase the number of peptide units to study the mechanisms of the spontaneous assembly of ChenW into oligomeric structural ensembles in the membrane. Density profiles (Figure 6C,D,G,H) showed water translocation and the transmembrane stable conformation of all peptides.

### 2.6. ChenW Peptide Exhibits Slight Haemolytic Activity

**Figure 7:**
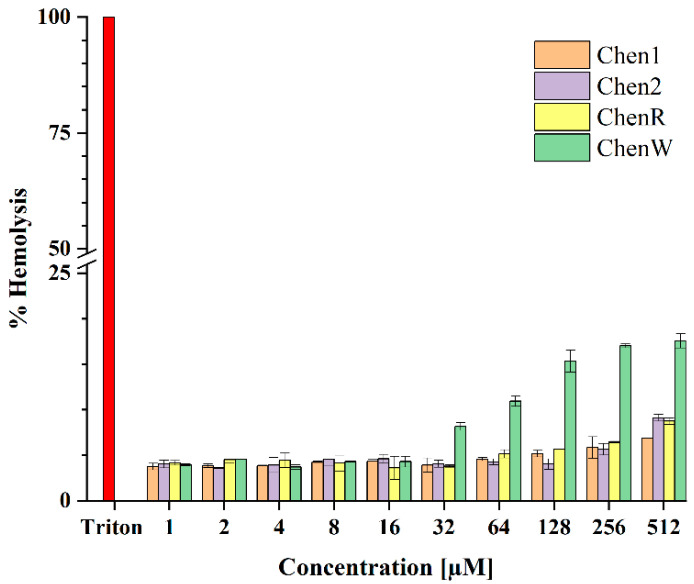
Evaluation of haemolytic activity of Chenopodin-derived peptides. Human red blood cells were incubated with synthetic peptides (1.0–512 µM) at 37 °C for 2 h. The level of haemolysis observed in the positive control (Triton X-100) was considered as 100% to calculate the levels in other samples. Data represent the mean ± S.D. (n = 3).

Both in silico and in vitro approaches were used to assess the peptide-induced haemotoxicity. In the first step, HLPred-Fuse and HAPPENN predictors were used to assess whether the Chenopodin-derived peptides possess any haemolytic activity. These tools suggested that ChenW is most likely to be haemolytic with predicted values of 0.5 and 0.01, respectively. However, Chen1, Chen2, and ChenR were not predicted to possess any haemolytic activity. Contrarily, the HemoPred classifier indicated the possible haemolytic action of Chen2 (Supplementary Material Tables S3–S5). Therefore, an in vitro haemolytic assay was performed. The data suggest that Chen peptides have a low haemolytic tendency, particularly at MIC concentrations tested. The peptides caused 3–18% red blood cell lysis at the concentrations tested. ChenW caused a larger proportion of haemolysis at concentrations over 32 µM (Figure 7). Therefore, this in vitro data is partially consistent with the bioinformatic screening performed using online tools, such as HLPred-Fuse and HAPPENN.

### 2.7. Chen2 and ChenR Peptides Display Antiviral Activities

**Figure 8:**
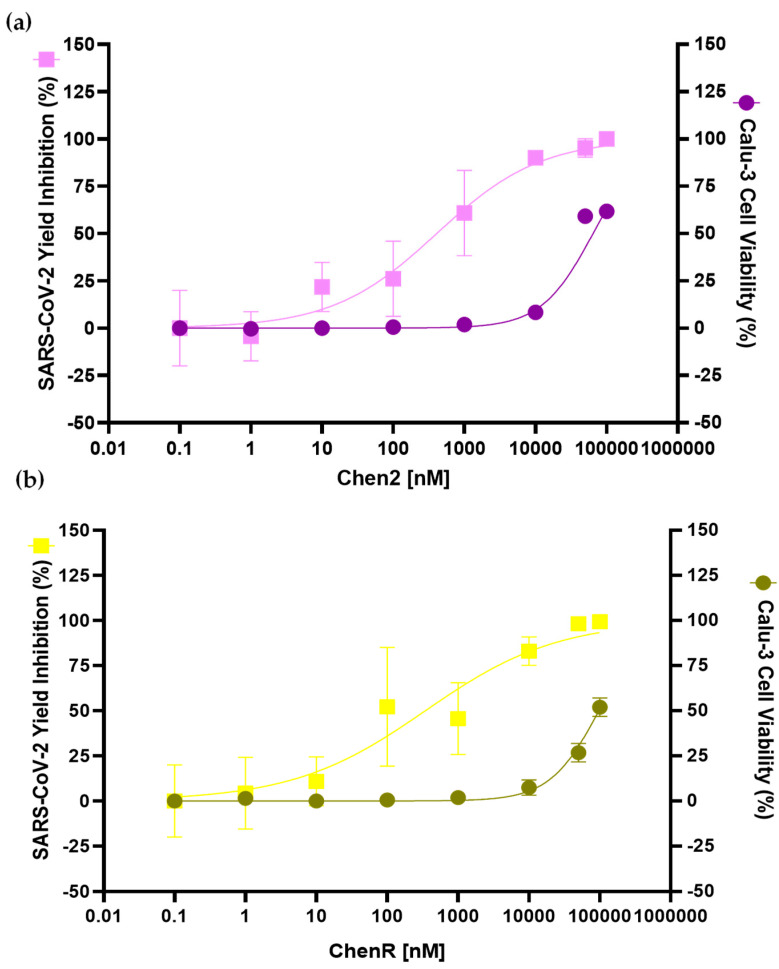
Antiviral activity of Chen2 (**a**) and ChenR (**b**) peptides in SARS-CoV-2-mediated infection of human pulmonary cells. SARS-CoV-2 yield inhibition (%), and cellular viability of Calu-3-infected cells are depicted in the graphs. Infected cells were incubated with a range of concentrations of each peptide. The SARS-CoV-2 yield inhibition (%) values were calculated in comparison to the negative control (infected, untreated cells). The cell viability assessed by MTT assay was expressed as % of mortality values and represents the means of eight repetitions with SD error bars. The SARS-CoV-2 Yield Inhibition (%) is a means of three repetitions with SD error bars.

Based on the results obtained from the antibacterial and haemolytic assays, Chen2 and ChenR (ChenW was not selected due to its slight haemolysis tendency) peptides were selected to test whether they possess any antiviral activity against SARS-CoV-2. The treatment of infected Calu-3 cells (submucosal gland cell line) with both Chen2 and ChenR caused a significant inhibition of SARS-CoV-2 replication at concentrations that preserved cellular viability (% of mortality < 10%) ranging from 0.0 nM to 10,000 nM (Figure 8). Chen2 presented higher antiviral activity compared to ChenR, showing an inhibition of SARS-CoV-2 replication superior to 75% in the 1000 nM concentration range. The EC_50_ (peptide concentration that inhibits 50% of the virus) values (Chen2: 407.8 nM; ChenR: 345.3 nM) and CC_50_ (cytotoxic concentration of the peptide that reduced cell viability to 50% values) (Chen2: 64.5 µM; ChenR: 97.4 µM) were obtained for the peptides. Overall, Chenopodin-derived peptides demonstrate a high selectivity index (SI), Chen2 SI = 158.1 and ChenR SI = 281.9.

## 3. Discussion

Peptides are attractive bioactive and biochemical scaffolds for the development of antibiotics [36,37]. From natural sources, such as plants [38] and venoms [39,40], the pharmaceutical market has gained several new prototypes, as well as better treatment options [41,42]. Advances in data-driven methods for predicting the bioactivity constitute a promising strategy to optimise the design of AMPs from plant sources by reducing their length, MIC, and host toxicity [43]. Here, we synthesised and characterised four structurally similar peptides based on the natural protein, Chenopodin from quinoa. The basic subunit of Chenopodin is rich in basic amino acid residues that are frequently found in typical AMP sequences [44]. Most of them have short lengths with hydrophobic cationic residues that form amphipathic structures [45]. AMPA software mapped a positive charge sequence made up of 14 amino acids (Chen1) in Chenopodin. This region was selected for in silico and in vitro screening. Additionally, we evaluated the in silico potential of a Chenopodin-based peptide of the same length (Chen2) that includes the last 10 residues of Chen1. Finally, two engineered peptides based on Chen1 were designed, replacing two amino acids with arginine (ChenR) or tryptophan (ChenW) residues. The computational approach of the AMPfun program suggested that the four computer-designed peptides possess antimicrobial activities. However, an in vitro screening of antibacterial activity revealed that Chen1 was not active against *S. aureus* and *E. coli* at the concentrations evaluated. Other investigations have also reported discrepancies between the in silico and in vitro analysis of peptides for their antimicrobial activities [46]. On the other hand, the other three peptides (Chen2, ChenR, and Chen W) showed activity against Gram-positive and Gram-negative bacteria, confirming the in silico predictions. They have an inhibitory effect in the micromolar range, similar to a broad-spectrum antibiotic (ampicillin) used as a control in our screening. The experimental study of new peptides predicted by in silico tools is fundamental, as it expands the data on the functionality of the peptides and provides valuable information for the improvement of the artificial intelligence methods used for antimicrobial screening.

Chen1 shared 10 amino acids with Chen2, and therefore, the difference in the activity may be due to the presence of asparagine (N) and glutamine (Q) located at the C-terminal, which are hydrophilic amino acids that can affect interactions with target membranes [47,48]. There are some examples in the literature that reported the presence of these amino acids compromises the antimicrobial activity of short peptides. For example, in the peptide P4bPP21NNN derived from *Moringa oleifera* seed, two proline (P) residues were replaced by asparagine (N), which is more hydrophilic and thus caused the antibacterial activity to be aborted [49]. Similarly, in the CT-K3K7 peptide, the amino acid, asparagine (N), was replaced by lysine (K), and it decreased its MIC from 12.5 μg/mL to 3.125 μg/mL [50]. Finally, Peña-Carrillo et al. [51] reported that the presence of asparagine in the pBmje peptide sequence may affect the antibacterial activity against *E. coli* and *S. aureus*. In summary, these investigations highlight how the presence and position of these types of amino acids modulate the antimicrobial activities of these peptides. Chen-2 reproduces an original fragment (AHSIIYGVRGRGRI) from Chenopodin, suggesting their determinant role in the antibacterial activity of this protein. The use of shorter molecules benefits the costs of synthesis and optimisation of new antibiotics. Chen2, ChenR, and ChenW peptides are more active against *E. coli* than *S. aureus*. Similarly, Porto et al. [43] reported the affinity of Guavanin-2 peptide with the *E. coli* membrane with a MIC of 6.25 μg/mL, while against *S. aureus,* it has a MIC of 100 μg/mL.

Engineering the primary structure of Chen1 produced bioactive sequences against both bacteria strains. Generally, the cationic property and hydrophobicity provides peptides with a higher potential to fight pathogenic organisms due to increasing the interactions with membranes [52]. Arginine and tryptophan have been pointed out as key elements to enhance the mode of action and beneficial effects of AMP, which involves predominantly membrane attraction, binding, insertion, and permeability [53,54]. The amino acid substitution employed here has also been successfully used in earlier studies [55,56].

The antimicrobial and haemolytic activity of Chenopodin-derived peptides are not coupled to each other. High toxicity and low selectivity leading to side effects have been critical obstacles to bringing peptide-based drugs to the pharmaceutical market [26]. In this study, in silico tools predict the non-toxicity or low haemolytic tendency of Chenopodin-derived peptides. These results were confirmed by in vitro assays. Peptides did not show erythrocyte membrane lysis as similar to their antimicrobial activity. ChenW showed the highest propensity for haemolysis, according to in silico results. Feng et al. [57] reported that peptides with a higher number of W residues are usually associated with a greater probability of damaging red blood cells. In another research, Staubitz et al. [58] showed that replacing five tryptophan residues with phenylalanine contributed to a decrease in the haemolytic activity of the peptide without compromising its antimicrobial activity.

Membranes are important biological and functional barriers for cells and promising drug targets [44]. The antibacterial properties of many AMPs lie in membrane-damage events [59,60]. The effect of peptides on *S. aureus* membranes was monitored by fluorescence microscopy using a dual DNA staining approach that combines permeable (DAPI) and non-permeable (PI) dyes [61,62]. Our results suggested that Chen2, ChenR, and ChenW peptides act by a membrane permeabilisation mechanism, manifested by increased red fluorescence. This is consistent with the blue fluorescence of DAPI and the absence of PI in untreated cells, since PI cannot penetrate intact bacterial membranes. These findings are comparable with studies, such as [63] by Liu et al., where GL-22 and GL-29 peptides showed damage against *S. aureus* by a membranolytic mechanism suggested by fluorescence microscopy. On the other hand, it has been observed that bacteria exposed to peptides tend to cluster together, a characteristic event previously described due to instability caused by the reduced membrane potential [64]. This biochemical phenomenon characterised by membrane lysis was clearly evidenced from the mechanistic point of view in our molecular dynamic simulations using membrane models of Gram-positive and Gram-negative bacteria. The formation of pores in bacterial membranes through the association of multiple copies of ChenW seems to be key to its antimicrobial action and is probably the mechanism used by the other cationic peptides.

Dual acting-peptides with antiviral and antibacterial actions have gained a lot of attention, especially during the COVID-19 pandemic [65,66,67]. Peptide repositioning has been converted into a shorter pathway to explore other benefits and find new applications [25,68,69]. Some peptides have been tested in vitro against SARS-CoV-2 [70,71,72]. Brilacidin acts at the viral entry, thus altering the integrity of the virus. This compound inhibited up to 61% of the infectious titer and was characterised as dose-dependent [73]. Other peptides such as gramicidin S (from *Bacillus brevis*) and melitin (from bee venom) have the potential to eliminate SARS-CoV-2 from Vero cells within 12 h with EC50 values of 1.571 µg/mL and 0.656 µg/mL, respectively [74]. Moreover, to be used for lung treatment in SARS diseases, peptides should be effective against SARS-CoV-2 and have a low cytotoxicity to pulmonary cells. In the present study, we demonstrated that Chen2 and ChenR have the capability to inhibit SARS-CoV-2 replication in human pulmonary cell culture with EC_50_ (407.8 nM and 345.3 nM, respectively) values substantially lower than those for CC_50_ (64.5 µM and 97.4 µM) values. These results corroborate the initial predictions of in silico tools (AMPfun and AI4AVP), suggesting the antiviral activities of Chenopodin-derived peptides. The underlying mechanisms of antiviral properties of these peptides remain to be elucidated. Taken together, our naturally available template-based peptide design approach identified that the primary structure of Chenopodin serves as a starting point for the further refinement and lead optimisation of new membrane-disrupting peptides with antibacterial and antiviral properties. Future studies will address the antiviral mode of action of bioactive Chenopodin-derived peptides towards SARS-CoV-2 and evaluate their serum stability.

## 4. Materials and Methods

### 4.1. Computer-Aided Design of Cationic Peptides Based on Chenopodin

#### Screening of Chenopodin Sequence: Prediction of Toxicity, Structure and Biological Activity

AMPA software (http://tcoffee.crg.cat/apps/ampa/do, accessed on 2 February 2023) [75] was used to screen the Chenopodin protein (UniProtKB—Q6Q384_CHEQI) for antibacterial candidate peptides for chemical synthesis. The antimicrobial region was selected as the source of peptide fragments, which were posteriorly examined using bioinformatics tools such as: AMPfun (http://fdblab.csie.ncu.edu.tw/AMPfun/run.html, accessed on 2 February 2023) [76] and AMP Scanner (www.ampscanner.com, accessed on 2 February 2023) [77] to predict antimicrobial activity; HLPpred-Fuse (http://thegleelab.org/HLPpred-Fuse/, accessed on 2 February 2023) [78], HAPPENN (https://research.timmons.eu/happenn, accessed on 2 February 2023) [79] and HemoPred (http://codes.bio/hemopred/, accessed on 2 February 2023) [80] to test for haemotoxicity; and AI4AVP (http://axp.iis.sinica.edu.tw/AI4AVP/, accessed on 2 February 2023) [81] for assessing the antiviral potential. Four novel peptides with high in silico antimicrobial potential were identified by a physicochemical-guided design and employed in the experimental phase. Briefly, two peptides, Chen1 (YGVRGRGRIQIVNA) and Chen2 (AHSIIYGVRGRGRI), mimic short molecular fragments of the parent protein (Chenopodin). The first one reproduces the amino acids between positions 72 and 87 in the sequence, while Chen2 has the same sequence found between 67 and 82. Two engineered cationic/hydrophobic peptides were designed based on Chen1 replacing amide side-chains amino acids with arginine (ChenR) or tryptophan (ChenW). Finally, the charge, hydrophobicity, isoelectric point (pI) and mass of the peptides were estimated using PepDraw (http://www.tulane.edu/~biochem/WW/PepDraw/, accessed on 2 February 2023). In addition, the secondary structure was investigated with I-TASSER and supplemented with information provided by PED2D (https://webs.iiitd.edu.in/raghava/pep2d/submit.html, accessed on 2 February 2023).

### 4.2. Chenopodin-Directed Peptide Synthesis, Purification, and Molecular Mass Determination

These computationally selected 14-mer peptides were obtained using the Fmoc solid-phase chemical synthesis technique with the Liberty Blue-automated microwave peptide synthesiser (CEM Corporation, Matthews, NC, USA) with >90% purity. The main solvent used was N, N’-dimethylformamide, and 0.192 g of Rink Amide Novabiochem resin, 0.52 mmol/g substitution at filling. After removing the protective groups, synthetic peptides were cleaved from the resin using 95% trifluoroacetic acid, 2.5% triisopropylsilane, and 2.5% water. The final products were washed in cold ethyl ether and freeze-dried for 24 h at −80 °C with 0.09 mT of pressure [51]. Purity was analysed by reverse-phase high-performance liquid chromatography (RP-HPLC), and peptides’ molecular weight was confirmed by matrix-assisted laser ionisation/desorption time-of-flight mass spectrometry (MALDI-TOF MS). The protocols for purity profiling and mass spectrometry characterisation were previously described in Peña-Carrillo et al. [51].

### 4.3. Biological Evaluation

#### 4.3.1. Peptide Bactericidality

Broth microdilution protocol previously employed by Peña-Carrillo et al. [51] was taken into account to determine the minimum inhibitory concentration against *Staphylococcus aureus* ATCC 25923 and *Escherichia coli* ATCC 25922. Briefly, lyophilised peptides were solubilised in dimethyl sulfoxide (DMSO). Each micro-organism reached the logarithmic phase and was diluted to 1 × 10^6^ CFU/mL. Then, 198 µL of microbial suspension in culture medium and 2 µL of the peptide solutions were dispensed into 96-well plates. Ten different peptide concentrations (1, 2, 4, 8, 16, 32, 64, 128, 256, 512 µM) were assessed. As a control, 2 µL of DMSO was used instead of the peptide, as well as 198 µL of the microbial culture media; for the negative control, Mueller Hinton Broth was used in the absence of microorganisms. Three independent experiments in triplicate were performed for each concentration. The plates were incubated at 37 °C for 18 h, and the inhibition of microbial growth was measured spectrophotometrically at 600 nm using a GloMax multiplex detection system (Promega, Madison, WI, USA). Finally, 10 µL from the MIC value wells were cultured on Mueller Hinton Agar. It was incubated at 37 °C for 18 h to identify the concentrations with no microbial growth that were considered as the Minimum Bactericidal Concentration (MBC). Ampicillin, a broad-spectrum antibiotic, was employed as a positive control and for comparison purposes in the screening of Chenopodin-derived peptides.

#### 4.3.2. Antiviral Activity and MTT Viability Assay

The antiviral analysis was performed using human lung epithelial cells Calu-3 (ATCC^®^-HTB-55™) cultured in Dulbecco’s modified Eagle medium (DMEM) and supplemented with 20% (*v*/*v*) fetal bovine serum, 1% (*v*/*v*) non-essential amino acids, 1% (*v*/*v*) penicillin/streptomycin, 2 mM Sodium Pyruvate, and 5 mM L-glutamine, and maintained at 37 °C and 5% CO_2_. SARS-CoV-2 strain SP02-BRA (Wuhan Hu-1) stock virus was titrated in Vero cells (ATCC^®^-CCL-81) and stored at −80 °C. The SARS-CoV-2 virus was kindly provided by Dr. Edison Durigon from Universidade de São Paulo. All in vitro screening involving the infectious virus was carried out in a BSL-3 facility under the control of Centro de Laboratórios Multiusuários - CELAM - in the Institute for Biological Sciences at Universidade Federal de Minas Gerais.

The antiviral properties of Chenopodin-derived peptides were determined after the infection of Calu-3 cells with the SARS-CoV-2 virus at moi = 0.1 and 48 h post-infection. The viral load (PFU/mL) yielded in each condition tested was determined using plaque assay in Vero cells. Calu-3 cells were seeded in 96-well plates at a density of 2 × 10^4^ Calu-3 cells two days before infection. After inoculation with SARS-CoV-2, the infected monolayers were then treated with an increasing concentration gradient of Chenopodin-derived peptides (0.1 nM, 1 nM, 10 nM, 100 nM, 1 µM, 10 µM, 50 µM, and 100 µM). The drug Nafamostat was used as a positive control. The supernatant from three of eight replicates for each treatment was collected and titrated to quantify the SARS-CoV-2 replication in each condition.

For the viability test, the MTT solution was added to each well of Calu-3 cells (500 µg/mL), infected or uninfected, and submitted to the same treatment with Chenopodin-derived peptides, as described above. MTT-treated monolayers were then incubated for 1 h at 37 °C, 5% CO_2_. After 1 h, the overlay media was discarded, and the formazan crystals were solubilised with DMSO. The absorbance was measured at 570 nm (MultiskanTM FC Microplate Photometer, Thermo Scientific Waltham, MA, USA) to report cell viability [82].

#### 4.3.3. Hemolytic Activity

Peptide-induced red blood cell membrane lysis was investigated using the approach outlined by Peña-Carrillo et al. [51]. Lyophilised aliquots of pure peptides were solubilised in DMSO to obtain the same concentrations previously used to evaluate antibacterial properties. Peptide solutions were incubated for 2 h at 37 °C with a 1:1 (*v*/*v*) dilution of 4% erythrocytes (O+), totalizing 200 µL. The cells were then dispersed in a 96-well plate after being centrifuged at 1000 rpm for 5 min. The percentage of hemolysis was measured using a GloMax multiplex detection system (Promega, Madison, WI, USA) at 550 nm. Three independent experiments were carried out in triplicate. Negative and positive controls were PBS and 2% (*v*/*v*) Triton X-100, respectively. The maximum percentage of hemolysis (100%) was defined as the absorbance monitored by the effect induced by Triton X-100. This was calculated using Equation (1).

(1) $$\%\;Hemolysis\;=\;\left(\frac{A-Aο}{A\chi -Aο}\right)$$

This formula includes the absorbance of the peptide (A), the absorbance of the negative control (Aο), and the absorbance of the positive control (Aχ).

### 4.4. Insights into the Mechanism of Action

#### 4.4.1. Fluorescence Microscopy to Assess Membranolytic Properties

The experimental protocol described by Valdivieso-Rivera et al. [83] was employed to visualise changes in bacterial cell permeability induced by Chenopodin-derived molecules. Peptide dilutions were made using MIC concentrations that had been determined previously and cultured with log-phase bacteria for 30 min before centrifugation at 3000× *g* for 15 min. The pellet was stained for 15 min in the dark at 0 °C with PI (120 mM) and DAPI (1 µM). Bacteria treated with 10% sodium dodecyl sulfate (SDS) were employed as the positive control, and microorganisms without peptide incubation as the negative control. Finally, the membrane damage was visualised using a Nikon Eclipse Ni microscope employing a combination of fluorescence and differential interference contrast (DIC). Fluorescent micrographic images were processed using ImageJ software.

#### 4.4.2. Molecular Dynamic Simulations

ChenW peptide was modelled with Build Biopolymer from Sequence tool on Maestro and exported as PDB. The peptide was relaxed on a system with a water solution; to allow the peptide to adopt a relaxed and free configuration, the system was built with Charmm-gui with TIP3 water molecules and 0.15 M NaCl. The molecular dynamics’ (MD) simulation was performed for 100 ns with Amber20, NPT ensemble, 310 K, FF19SB/TIP3P/GAFF2 force fields, cutoff radius 12 Å for short-range Coulombic interaction, integration time step 2 fs, and no restraints. After the relaxation simulation, we built six new systems using Charmm-ui, three systems with PVPE:PVPG:PVCL2 (ratio 75:20:5) to simulate the Gram-negative inner membrane and three different peptide configurations, a single extended peptide crossing the membrane, six parallel peptides, and six antiparallel peptides. The other three systems were built to simulate a Gram-positive inner membrane using POPE:POPG (ratio 75:25) and the same peptide configurations as the last three systems. All systems had a 0.15 M NaCl ion concentration. The systems were defined from the inner layer of the membrane based on the fluorescence microscopy image assessment that suggests the inner membrane as a potential site of action for Chenopodin-derived peptides. Many conventional antibiotics also act with a mechanism based on the disruption of the inner membrane.

Molecular dynamics were performed with Amber20 for each of the six systems, and equilibrium steps followed the predefined protocol written by Charmm-Gui for Amber, applying restraints of 10 kcal × mol^−1^ × Å^−2^ to the peptides and decreasing them to 0.1 before the production MD. For production simulations, 1 μs MDs were performed using the NPT ensemble, 310 K, FF19SB/lipid21/TIP3P/GAFF2 force fields, integration time step 2 fs, cutoff radius 12 Å, and 10 Å for short range Coulombic interaction for six peptide systems and single peptide system, respectively. Density profiles were calculated through the whole molecular simulations using the VMD density profile plugin, considering the atomic mass of heavy atoms and ignoring hydrogens.

### 4.5. Statistical Analysis

The results were analysed by a two-way analysis of variance (ANOVA) and Tukey’s test, using the OriginPro 9.0 Software (Origin Lab, Northampton, MA, USA). The level of significance was established with *p* < 0.05. For the antiviral analysis, the EC50 values were calculated using GraphPad Prism 8 as the concentration at which there was a 50% decrease in viral replication in comparison with untread controls (0% inhibition). Curves were fitted based on a four-parameter non-linear regression.

## 5. Conclusions

In conclusion, we identified a sequence (Chen2) that reproduces the antibacterial effect of Chenopodin. Engineered peptides were also active against *E. coli* and *S. aureus*, demonstrating the utility of amino acid substitution to improve the activity of therapeutic peptides. The antibacterial action of these Chenopodin-inspired molecules involves changes in cell permeability. Chen2 and Chen R are also promising antiviral agents. Overall, this investigation reveals clues for engineering peptides and opens new research avenues to explore the biotherapeutic potential of vegetal proteins as privileged scaffolds and rich sources of shorter dual-acting molecules with membrane-damaging mechanisms.

## Figures and Tables

**Table 1:** Physicochemical characteristics of the Chenopodin-derived peptides. Isoelectric point (pI), charge, and hydrophobicity of these peptides were predicted using PepDraw. Peptides are amidated, and the C-terminal ends were considered for estimating these properties.

Peptides	Length (aa)	pI	Charge	Hydrophobicity (Kcal∙mol^−1^)	Mass (Da)
Chen1	14	12.79	+4	+15.03	1556.89
Chen2	14	12.79	+4	+15.54	1552.90
ChenR	14	13.09	+6	+17.03	1626.99
ChenW	14	12.79	+4	+9.23	1686.95

**Table 2:** Minimum inhibitory concentration (MIC) and minimum bactericidal concentration (MBC) of synthetic Chenopodin-derived peptides. (-) No effects were observed in the concentrations tested. The same result was obtained in three independent experiments, each time in triplicate.

Peptides/Antibiotic	MIC (µM)		MBC (µM)	
	*E. coli*	*S. aureus*	*E. coli*	*S. aureus*
Chen1	-	-	-	-
Chen2	64	128	128	128
ChenR	16	128	32	256
ChenW	8	8	8	8
Ampicillin	16	2	64	8

## Data Availability

All data supporting the findings of this study are available within the paper and its Supplementary Material.

## Supplementary Materials

The following supporting information can be downloaded at: https://www.mdpi.com/article/10.3390/antibiotics13010078/s1, Table S1: Prediction of the antimicrobial activity of Chenopodin-derived peptides using Ampfun software. Table S2: Prediction of the antimicrobial activity of Chenopodin-derived peptides by AMP Scanner software. Table S3: Prediction of the haemotoxicity of peptides derived from Chenopodin protein by HAPPENN. Table S4: Prediction of the haemotoxicity of Chenopodin-derived peptides by Hlppred-fuse. Table S5: Prediction of the haemotoxicity of Chenopodin-derived peptides by HemoPred. Table S6: Prediction of the potential antiviral effect of Chenopodin-derived peptides by AI4AVP. Table S7: Prediction of the secondary structure of Chenopodin-derived peptides by PED2D. Figure S1: RP-HPLC chromatograms of Chenopodin-derived peptides. Purified synthetic peptides (a) Chen1, (b) Chen2, (c) ChenR and (d) ChenW elute between 26 and 29 min.
