# Supplementary material for: Antibacterial and Antiviral Properties of Chenopodin-Derived Synthetic Peptides
Source: Antibiotics (Basel). 2024 Jan 14;13(1):78. doi: 10.3390/antibiotics13010078 (PMC10812719; doi:10.3390/antibiotics13010078)
Supplement: Supplementary file 1 [file antibiotics-13-00078-s001.zip › antibiotics-2772881-supplementary.pdf]

Supplementary Material

Antibacterial and antiviral properties of Chenopodin-derived synthetic peptides

Table S1: Prediction of the antimicrobial activity of Chenopodin-derived peptides using Ampfun software.

ID	AMP	Antiparasitic	Antiviral	Anticancer	Targeting mammals	Antifugal	Gram + bacteria	Gram - bacteria
Chen1	0.9633	0.1182	0.625	0.1581	0.0667	0.4678	0.675	0.5362
Chen2	0.997	0.0545	0.5833	0.1925	0.0667	0.7167	0.8083	0.6232
ChenR	0.9884	0.1	0.5667	0.1648	0.1	0.5763	0.7583	0.5362
ChenW	0.9829	0.0636	0.5917	0.1058	0.0667	0.3664	0.6833	0.7213

Table S2: Prediction of the antimicrobial activity of Chenopodin-derived peptides by AMP Scanner (www.ampscanner.com.) software.

ID	Predicted Class	Prediction Probability*
**Chen1**	Non-AMP	0.1207
**Chen2**	AMP	0.9718
**ChenR**	AMP	0.9358
**ChenW**	AMP	0.9872

***> 0.5 = Predicted AMP**

Table S3: Prediction of the haemotoxicity of peptides derived from Chenopodin protein by HAPPENN.

ID	Predicted probability
**Chen1**	0.007
**Chen2**	0.003
**ChenR**	0.004
**ChenW**	0.010

Table S4: Prediction of the haemotoxicity of Chenopodin-derived peptides by Hlppred-fuse.

ID	High or low	Predicted probability of High
**Chen1**	**Low**	0.341666666667
**Chen2**	**Low**	0.384375
**ChenR**	**Low**	0.384375

**ChenW**	**High**	0.539583333333

Table S5: Prediction of the haemotoxicity of Chenopodin-derived peptides by HemoPred.

ID	Status
**Chen1**	Non-hemolytic
**Chen2**	Hemolytic
**ChenR**	Non-hemolytic
**ChenW**	Non-hemolytic

Table S6: Prediction of the potential antiviral effect of Chenopodin-derived peptides by AI4AVP.

ID	Score	Prediction Results*
**Chen1**	0.981	YES
**Chen2**	0.918	YES
**ChenR**	0.991	YES
**ChenW**	0.996	YES

*Yes: Peptide is classified as an antiviral peptide.

Table S7: Prediction of the secondary structure of Chenopodin-derived peptides by PED2D.

ID	Secondary Structure	Helix %age(H)	Sheet %age(E)	Coil %age(C)
**Chen1**	CCCCCCCCEEHCCC	7.14	14.29	78.57
**Chen2**	CCEECEECECCCCC	0.00	35.71	64.29
**ChenR**	CCCCCCCCEECCCC	0.00	14.29	85.71
**ChenW**	CCCCCCCEEEECEC	0.00	35.71	64.29

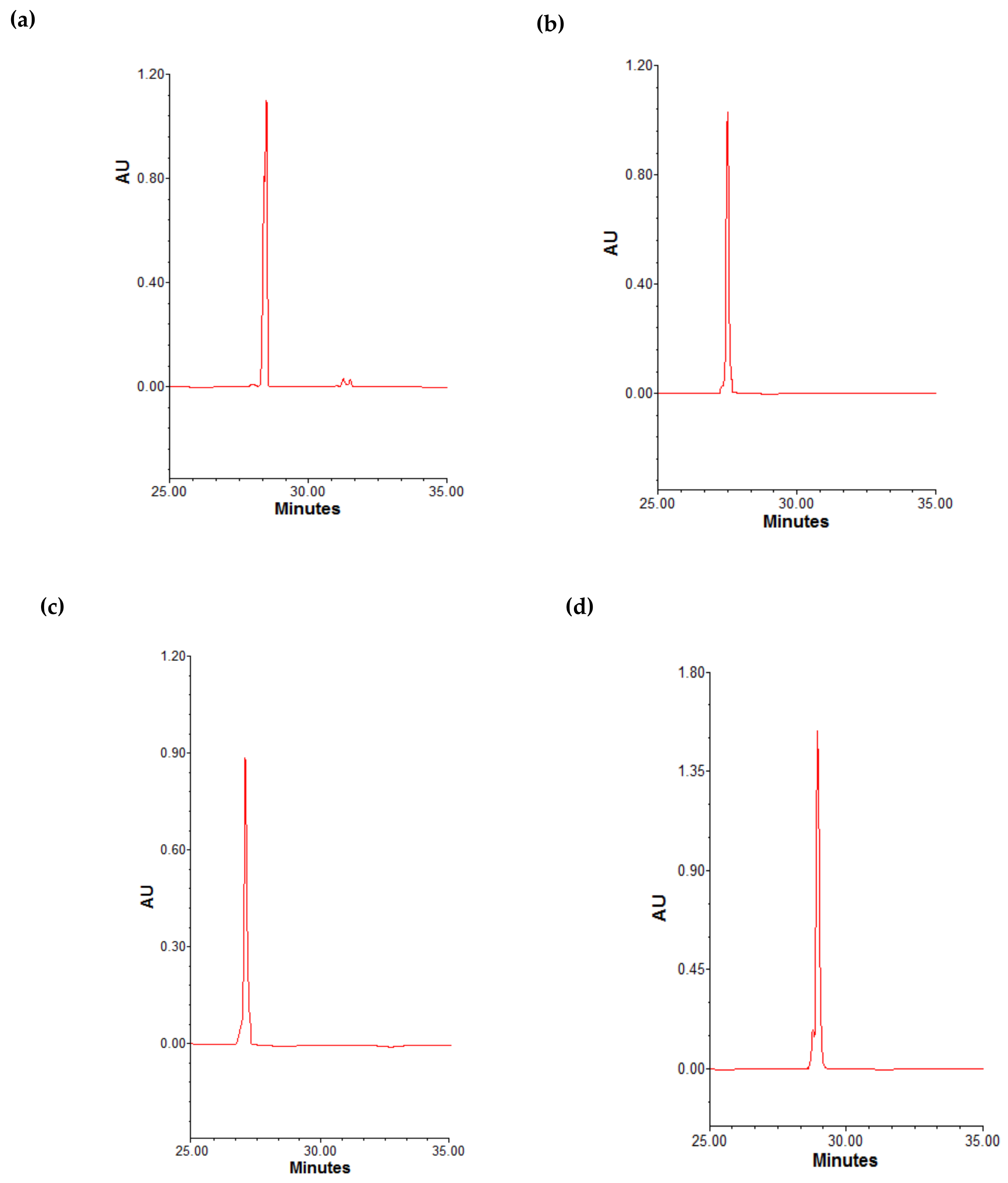

Figure S1: RP-HPLC chromatograms of Chenopodin-derived peptides. Purified synthetic peptides **(a)** Chen1, **(b)** Chen2, **(c)** ChenR and **(d)** ChenW elute between 26 and 29 min.
